# Young people’s experiences of informal kinship care in Luwero, Uganda

**DOI:** 10.1016/j.childyouth.2024.107527

**Published:** 2024-04

**Authors:** Simone Datzberger, Jenny Parkes, Amiya Bhatia, Rehema Nagawa, Joan Ritar Kasidi, Brian Junior Musenze, Karen Devries

**Affiliations:** a University College London, UCL Institute of Education, United Kingdom; b Medical Research Council / Uganda Virus Research Institute & LSHTM Uganda Research Unit, Uganda; c University of Oxford, Department of Social Policy and Intervention, United Kingdom; d Makerere Institute of Social Research, Uganda; e London School of Hygiene & Tropical Medicine (LSHTM), United Kingdom

**Keywords:** Kinship care, Uganda, Young people's perspectives, Children's wellbeing, Social policy

## Abstract

There is strong evidence that outside parental care, informal kinship care is the most practiced, sustainable and affordable form of childcare in SSA (sub-Saharan Africa). As a longstanding cultural tradition, informal kinship care embraces childcare as the responsibility of all extended family members, and often the wider community. However, over the past decades, informal kinship care has become gradually strained by political, economic and social conditions, such as: legacies of colonialism, increasing levels of poverty and inequality, instability, or infectious diseases. Drawing on qualitative biographical data from the Contexts of Violence in Adolescence Cohort Study (CoVAC) (2018–2022), we examine and showcase how practices of informal kinship care are perceived and experienced by young people. Doing so, we pay attention to the political, economic and social environment affecting informal kinship care in Uganda. Young people’s accounts point to four main features of their experiences of informal kinship care, that relate to: 1) mitigating poverty; 2) ensuring continuation of schooling and/or income generation; 3) generating and navigating family disputes and ruptures; 4) posing and moderating threats to safety and security of children. In analysing young people’s narratives, we critically reflect on implications for research and practice to support informal childcare. We argue that informal kinship care remains a vital cultural asset and an important alternative to forms of residential or institutional care in Uganda. This has implications for health, social protection, education programming and policy for children. Culturally sensitive and context-specific approaches, informed by the perspectives and lived experiences of young people, are needed to properly support children and young people in informal kinship care.

## Highlights

• Informal kinship care is a vital cultural asset and important alternative to forms of residential or institutional care.
• Informal kinship care occurs within the legacies of colonialism, strained by specific social, political and economic contexts.
• Informal kinship care networks are essential for children from low socio-economic backgrounds.
• Informal kinship care challenges western notions of family units and caregiving which prevail in global health programmes.
• Context-specific and meaningful understanding of children’s care and living arrangements is needed in programming and policy.

## 1 Introduction

Kinship care remains one of the most viable and sustainable forms of childcare outside of parental care in SSA (sub-Saharan Africa) (Martin and Zulaika, 2016, Ariyo et al., 2019, Mann and Delap, 2020). It is deeply rooted in pre-colonial cultural norms, practices and values, considering childcare as the responsibility of all family members, if not wider community (Assim, 2013, Petrowski et al., 2017). Uganda presents an interesting case as it has a higher prevalence of children living in informal kinship care than most other countries in the East African region (Zulaika and Martin, 2015). National household data from 2011 showed that 17 % of children (aged 0–14) did not live with their biological parents, out of which 97 % lived in informal kinship care with 91 % still having one parent and 69.4 % still having both parents alive (Martin and Zulaika, 2016). Ugandan children not in parental care tend to mainly live with one or both grandparents (55 %), followed by aunt and/or uncle (20 %), other relative(s) (12 %), sibling(s) (5 %), or not biologically related caregiver(s) (4 %). Only 3 % are officially adopted or fostered (Zulaika and Martin, 2015).

As we will show in this paper, evidence and insights from informal kinship care in Uganda, can have important implications for wider global debates and initiatives on children’s care, highlighting the need to refocus attention from “institutionalizing” children to the potential of informal systems of childcare and protection (U.N. General Assembly, 2010, Martin and Zulaika, 2016, Mann and Delap, 2020). This further extends to strategies and aid from national and international development actors, to provide more targeted and culturally sensitive and context-specific support reaching children in informal kinship care and supporting their extended family networks. This is in particular important, considering that raising children in different households by various family members has increasingly become also a necessity for caregivers to cope with mounting economic, social, environmental, health-related or political pressures (Chirwa, 2002, Bray and Dawes, 2016). The initial purpose, cultural tradition and nature of kinship care in SSA has changed significantly in recent decades to adapt to challenges intensified by colonization, globalization, urbanization, climate change, conflict or financial shocks. The recent COVID pandemic has further shown the importance of kinship care during prolonged periods of school closures and multiple lockdowns, while at the same time putting a lot of stress on extended family members with a sudden increase in caretaking responsibilities, as illustrated in the case of Uganda (Parkes et al., 2020).

There has been a growing interest by scholars in studying the changing nature and purpose of kinship care in SSA since the late 1970s. Initially a subject of interest to demographers and anthropologists (e.g.: Isiugo-Abanihe, 1985, Leinaweaver, 2014, Mbugua, 2014, Brown et al., 2020), research in public health has also started to examine the impact of kinship care on children’s wellbeing (e.g.: Ampiah and Adu-Yeboah, 2009, Roby et al., 2014, Beck et al., 2015, Bray and Dawes, 2016, Lachaud et al., 2016, Ariyo et al., 2019, Mann and Delap, 2020, UNICEF, 2020). There has also been some focus on the social capital of kinship structures (e.g.: Muruthi, Dolbin-MacNab and Jarrott, 2021) and on how taking care of various children affects caregivers themselves, in particular grandparents (e.g.: Nahemow, 1979, Chirwa, 2002, Martin and Zulaika, 2016, Ddumba-Nyanzi and Li, 2018, Schrijner and Smits, 2018). With only a few exceptions (see: Notermans, 2008, O’Kane, 2020), far less attention has been given to qualitative and biographical methods to analyse how young people themselves perceive their kinship care experience, and how they benefit, cope, adapt and adjust to their changing situation and family relationships. Against this backdrop, we are interested in the following questions: *How do young people experience and perceive kinship care?* And consequently: *What can we learn from their accounts to provide targeted and context-specific support to informal kinship care networks?* In discussing young people’s biographical narratives, we draw on the accounts of 17 young people (11 female and 6 male), who all experienced kinship care during the ages 0–18 and who participated in the qualitative component of our mixed methods study CoVAC (Contexts of Violence in Adolescence Cohort Study 2018–2022) (Devries et al., 2020). Over the course of the study, we interviewed each participant seven times (see Annex) and progressively constructed biographical narratives for each participant.

We start with an overview of kinship care in the context of SSA, followed by a short discussion of existing research addressing children’s wellbeing in kinship care. We continue with a section on the political, economic and social conditions affecting kinship care in Uganda. As explained in our methods section, our biographical narratives and qualitative data from seven rounds of research pointed to four main characteristics shaping our cohort’s kinship care experience. We subsequently discuss and analyse young people’s accounts and experiences regarding these characteristics and conclude by critically reflecting on implications for future research, global debates on childcare and development assistance and practice.

## 2 The changing nature of informal kinship care in SSA

For the purpose of this paper, we broadly understand *informal kinship care* (‘*okulabirira ab’oluganda*’ in Luganda), as (definition amended from: Ddumba-Nyanzi and Li, 2018, p. 61):

*“Any private arrangement provided in a family environment, whereby the child is living with and looked after by extended family or close friends of the family known to the child in their individual capacity, at the initiative of the child, his or her parents, or another person, without this arrangement having been ordered by an administrative or judicial authority or a duly accredited body.”*

This residential arrangement can be ongoing, short- or long-term, indefinite or set for a specific period of time. It is thus *not officially controlled* by any kind of state authority (such as foster care, adoption, which can be formal kinship care by relatives or formal care by non-relatives). As shown in our methods section (Table 1), we follow the definition above, and do not consider young people as being in informal kinship care when they live with both or one of their biological parents but receive financial or any other support from an extended family member or close family friend. At the same time, we acknowledge that informal kinship care is a term rooted in colonial definitions that privilege certain forms of ‘formal’ care arrangements and ideas of nuclear or specific family units as central to how children should grow up. With this paper we want to challenge some of these colonial prejudices.

As a traditional form of childcare and a social support system, kinship care has been practiced in the SSA region for centuries, dating back to pre-colonial times. An individual child is seen as being deeply connected to all extended relatives through a broad network of blood relations and not just to their nuclear family. Parenting is consequently interlinked with existing structures of kinship relations, with different roles and responsibilities among adults to raise ‘their’ children. Traditionally, this would involve biological parents providing for children’s necessities (such as school fees or food); whereas sex education tends to be seen as the responsibility of uncles and aunts; or teaching young people respect for elders is considered a task for all family members and if not the community (Mbugua, 2014, Bray and Dawes, 2016). In the particular case of Uganda, informal kinship care was also a means to create a bond among non-blood related families. In the Baganda culture^1^ it used to be a common practice to look after children from a different clan, to strengthen social networks, social capital and social relations (Nahemow, 1979). Despite the distortion of traditional family systems during colonial times, informal kinship care remains a practice strongly embedded in SSA, and not just in Uganda (Martin and Zulaika, 2016, Mann and Delap, 2020). As a tradition, it challenges western conceptualizations and modern notions of parenthood, by acknowledging the boundaries and limits of (biological) parental care and the nuclear family. This further extends to the importance placed on reciprocity within extended family networks (Matovu, Rankin and Wallhagen, 2020). There is an underlying expectation, especially from SSA grandparents, that children contribute practically to the kinship care household (e.g.: helping with chores or other tasks), or that biological parents provide financial compensation for their efforts (Matovu, Rankin and Wallhagen, 2020). The wider and more cohesive the network, the greater its social adaptive capacity to deal with crises such as caring for children whose biological parents are absent for various reasons, due to poverty, conflict, illness or death (Chirwa, 2002, Bray and Dawes, 2016). From this perspective, kinship care is much more than a network of extended family members that raise their children in unison. In the best case, it provides a solid and reliable social support system for children and older generations alike (Nahemow, 1979, Matovu et al., 2020). In the worst case, it affects children’s well-being negatively, in instances when caregivers are either unable or unwilling to provide for their basic needs or, when children become subject to abuse and neglect (Mann and Delap, 2020).

## 3 Kinship care and the wellbeing of children

Global studies drawing on national household data from multiple country-contexts reveal that children raised in a range of family environments, such as extended biological family networks and foster- or adoptive families, generally fare better than children raised in institutional care in terms of physical, cognitive, and socio-emotional development (Johnson and Gunnar, 2011, Schoenmaker et al., 2014, Martin and Zulaika, 2016). However, the main challenge in establishing a specific relationship between informal kinship care in SSA and the wellbeing of children, is that we can neither observe, compare or measure what would have happened to an individual child if it remained with its biological parents (Beck et al., 2015). In general, research examining the relationship between children's welfare and kinship care reveals varied and complex results, influenced by factors such as context, the specific indicators used to define 'wellbeing', and the outcomes being measured, such as health, education, or physical and emotional wellbeing. There is some evidence that children in kinship care may have poorer health outcomes compared to children in other care settings (Ariyo, Mortelmans and Wouters, 2019). This, according to one study carried out in Uganda, can be attributed to the low socio-economic status of the caregiving families (Roby, Shaw and George, 2014). In systematically reviewing 23 studies from SSA, Ariyo, Mortelmans and Wouters (2019) found that factors which tend to positively influence children’s wellbeing in kinship care include: relatedness to the caregiver; socio-economic status of the caregiver (i.e. the overall quality of kinship care depends on the wealth of the family); age and gender of the child (older girls above the age 10 seem to be most disadvantaged) and the reasons for kinship care (children who are in extended family care to strengthen social ties and relationships fare better than children who are in non-parental care due to crisis). Outside of parental care, several studies have claimed that grandparents provide the best kinship care. Children under their care were more likely to be enrolled in primary school compared to other extended family members who acted as caregivers (Lachaud, LeGrand and Kobiané, 2016) and less likely to exhibit stunted growth (Schrijner and Smits, 2018). Other relatives (such as aunts or uncles) were found to be more likely to support secondary education (Lachaud, LeGrand and Kobiané, 2016). In a study conducted in Senegal, kinship care seems to be favourable for children in regard to schooling, which may be linked to parents’ motive to send they children away for the purpose of keeping them enrolled in school (Beck et al., 2015). The same study also found that fostered girls in Senegal did not spend more time on domestic chores than their host sisters, contrary to a different study carried out in Uganda, which found higher levels of domestic work by kin children (Roby, Shaw and George, 2014).

The above suggests that research on children in kinship care should be generally interpreted with some caution as studies use different methodologies, indicators, sample sizes and are conducted in different contexts and periods. However, scholars appear to generally agree that outside parental care, kinship care is the most sustainable and affordable form of childcare in SSA, and a much better alternative for children than institutional care. There is also a broad consensus that children in kinship care are largely treated on a fair basis relative to their host siblings and have the same safety indicators than children in parental care (Ariyo et al., 2019, Mann and Delap, 2020). However, scholarship also points to significant gaps that restrict our knowledge on kinship care, such as the need for more qualitative study designs focusing on children’s and young people’s perspectives (Cuddeback, 2004, Notermans, 2008, Ariyo et al., 2019, Mann and Delap, 2020). Scholars also suggest approaching kinship care in study designs as a dynamic experience, which is shaped by fluidity and adaptability of families and children with changing power dynamics, relationships, norms, circumstances and emotions (Notermans, 2008). Our contribution to these gaps in research is twofold: First, we draw on qualitative data collected over five years (see methods section). Second, our analysis is centred around young people’s viewpoints, taking the fluid, changing nature of their circumstances into account. We are thus interested in how young people themselves experience, cope with, manoeuvre and negotiate through different facets and changes in their kinship care experiences and living arrangements. Doing so, we pay attention to the changing political, economic and social conditions that shape young people’s experiences of kinship care.

## 4 Political, economic and social conditions affecting informal kinship care in Uganda

Our study was conducted in a context of changing informal kinship care arrangements. Below we outline past and present political, economic and social conditions affecting informal kinship care in Uganda. An understanding of how these conditions influence childcare in Uganda is important, as the current characteristics of informal kinship care cannot be detached from the legacies of colonialism, followed by neoliberal economic policies, structural adjustment programmes, conflicts, capitalism, corruption and persisting discriminatory global power structures.

### 4.1 Political environment

The typical pre-colonial African family was characterized by extended family networks encompassing wide-ranging degrees of kinship. These traditional family relations were significantly reshaped and distorted during British colonialism (Mamdani, 1996). After independence, a bifurcated post-colonial state emerged (Ekeh, 1975), in which customary law and practices run in parallel or interfere with official family law. These have been criticised by many Ugandan feminists for supporting heteropatriarchal family structures (Tamale, 2020 pp. 321–339). For example, cohabiting couples in Uganda are traditionally considered as married, but without a formal ceremony and registration their marriage is not recognized by official law. Consequently, cohabiting couples are not protected by the law in Uganda, disadvantaging in particular women (and their children) as they do not qualify for any legal benefits that come with marital status (e.g.: health insurance, inheritance rights, etc.) (Tamale, 2020, p. 304). The tensions between official and traditional/customary law are also salient when it comes to informal kinship care. Even though Uganda’s NFAC (National Framework for Alternative Care) 2012 recognizes informal kinship care as one of the alternative care options and de facto responsibility of informal carers for the child, it is not officially recognized by Ugandan authorities (Ddumba-Nyanzi and Li, 2018). Compared to institutional, adoption or foster care, informal kinship care continues to be the least systematically recorded, monitored or supported care option in Uganda – despite its vast prevalence. An assessment of Ugandan’s alternative care for children, concludes that (Ddumba-Nyanzi and Li, 2018, p. 31): “Children in informal kinship care and their caregivers may be assisted within the broader child protection system, however, they are not exclusively targeted for government and nongovernmental social support and counselling services. There are no specific mechanisms to assess carers’ and children’s needs, in terms of protection and support, or to ensure that informal kinship caregivers have access to available services and benefits”. The same assessment further highlights that there are currently no staff with defined responsibilities to monitor informal kinship care placements.

### 4.2 Economic environment

Ugandan caregivers are affected by extremely difficult economic conditions and poverty. Recent figures estimate that 42.1 % of all Ugandans live in multidimensional poverty (OPHI, 2022). Already five years prior to the pandemic, Uganda’s GDP (Gross Domestic Product) per capita growth halved to 1.1 % on average per year.^2^ The COVID pandemic has further aggravated the depth and severity of poverty in Uganda, in rural as well as in urban areas (World Bank, 2022). Child labour (aged 5–17) increased significantly from 13.7 % in 2016/17 to 17.5 % in 2019/20, with more males (20 %) than females (15 %) engaged in labour (UBOS, 2021, p. 84). Recommended strategies to strengthen Uganda’s Human Capital through education are challenged, among others, by caregivers’ inability to pay for school fees and consequently high school drop-out rates; what is being taught in schools and how education is governed and redistributed (Datzberger, 2018). A child who currently starts schooling at the age of 4 is only expected to complete 6.8 years of schooling by their 18th birthday, compared to the SSA average of 8.3. However, their actual years of learning are 4.3, with 2.5 years considered “wasted” due to the poor quality of education.^3^ The prolonged closure of schools during COVID has further aggravated the situation (Datzberger and Parkes, 2021, Datzberger et al., 2022). There is broad consensus in scholarship that children from wealthier households experience better wellbeing outcomes, than those from poorer households (Cooper and Stewart, 2021). This further extends to children in informal kinship care (Roby, Shaw and George, 2014). A recent study from central Uganda also showcases that the wealth of a family household has strong implications on the quality of childcare arrangements and children’s overall wellbeing (Nankinga et al., 2022).

### 4.3 Social environment

As noted earlier, Ugandan family arrangements were significantly reshaped and transformed by colonialism – in particular through the emergence of a new domesticity and the imposition of Western notions of household organization. Since independence, the social fabric of Ugandan families has again changed significantly. Various conflicts, the AIDS crisis of the 1990 s, and the recent COVID pandemic put a lot of pressure on and led to further fragmentation of extended families (Matovu, Rankin and Wallhagen, 2020). The average Ugandan household currently consists of five family members (UBOS, 2021), thereby increasingly resembling nuclear and western family arrangements. At the same time, it is striking, that despite these developments, informal kinship care continued to persist (Tamale, 2020, p 303). One implication of this is that children are no longer looked after by various extended family members in the same household, but rather have to move to a different household setting and environment (Martin and Zulaika, 2016).

Notably, gender plays an important role in how childcare arrangements can increase social injustices. Globally, many caregivers struggle to balance their responsibilities for childcare and income generating activities, with a disproportionate burden placed on women (Gromada, Richardson and Rees, 2020). Childcare in Uganda remains predominantly a women’s job. Women dedicate on average 10 h more to unpaid care work for children aged 5 or older per week than their male counterparts (UBOS, 2021, p. 90). With additional household tasks added, women and girls (aged 15 + ) spend 14.6 % of their time on unpaid care and domestic work, compared to 8.8 % spent by men.^4^ Female-headed households also have higher rates of multidimensional poverty, with 50 % compared to 39 % respectively for male-headed households (OPHI, 2022, p.25). However, one study found that children have significantly higher wellbeing when looked after by a female than male caregiver (Nankinga et al., 2022).

As we have shown in this section, informal kinship care is gradually strained by increasing levels of poverty and inequality, political and economic instability, armed conflict or infectious diseases (Assim, 2013, Ariyo et al., 2019). Families in Uganda face many intersecting challenges in varying degrees, ranging from poverty, unemployment, a general lack of opportunities, low educational attainment, a rapidly growing population, inter-generational power imbalances, persisting gender inequalities, illegal environmental resource exploitation, climatic changes or human rights abuses (Lubaale, 2019). To borrow Tamale’s words (2020, p. 335): “Todays monolithic heteropatriarchal families/marriages do little to address the realities on the ground.”.

## 5 Methods

Our study was conducted as part of a broader research project: Contexts of Violence in Adolescence Cohort Study (CoVAC). CoVAC is a mixed methodology cohort study that aims to build understanding on how family, peer, school and community contexts affect young people’s experiences of violence in adolescence and early adulthood (Devries et al., 2020). It includes epidemiological data collection at 3 time points, and a qualitative component, with fieldwork for 2–3 months each year from 2018 to 2022 in the Luwero District of Uganda. In total 36 young people, aged 13–17 years (in 2018), are the core participants in the qualitative research. These core participants were purposely selected from the project’s quantitative cohort of 3431 young people, based on their responses in our wave 1 epidemiological survey in 2014 and prior agreement to be contacted again. The qualitative sample includes equal numbers of girls and boys, from rural and urban communities, and with varying experiences of violence (more, or less severe). Each core participant was assigned a ‘key’ researcher, who is Ugandan, engages with them exclusively in the local language (Luganda), and where possible same sex (and always same sex for female participants), enabling good research relationships to be sustained over time. Carefully nurturing relationships of trust between the researchers and study participants has helped us to work with the same cohort of young people over the past five years.

Our qualitative data stems from 7 rounds of interviews and other encounters with the core participants since 2018 (see Annex). Each round helped us, to establish young people’s biographical narratives carefully and thoroughly from birth onward, to better understand their childhood experiences, to gain deeper insights of their lives and to probe past accounts and narratives. For this paper we draw on data from 17 (11 female, 6 male) of the core participants, who reported living for extended periods with extended family or friends, and away from parents when they were below the age of 18 (see Table 1). In a few instances, we included young people’s reflection of their informal kinship care experience after they had turned 18, showcasing that extended family support does not end once a child attained legal age.

One of our core methods has been to organise semi-structured interviews around a ‘river of life’. This meant young people co-constructed biographical narratives from their birth in a series of conversations with the researcher, using the metaphor of a river (with, for example, rocks symbolizing barriers, or flowers symbolizing happy moments) to draw and reflect on events, experiences, and emotions over time. At each round of data collection, young people extended their ‘rivers’, reflecting on events or including past experiences they may have missed in previous conversations. This method allowed us to slowly establish a relationship of trust between the key researcher and the young person we interviewed. It also helped us to avoid direct questions about difficult personal experiences. However, if the young person chose to narrate such experiences, we could gently probe for circumstances, support networks (i.e. kinship arrangements), and change over time (such as change of households and caregiver arrangements). This made it possible to probe around past accounts of significant events, experiences, norms and practices, or relationships. Overall, our analysis examined both, retrospective accounts and the changing circumstances of core participants during childhood and through adolescence.

Data were translated (from Luganda to English) and transcribed by the research team, and then coded using NVivo. Our analysis developed through regular team discussions on emerging findings alongside analysis of the coded data by thematic areas. The composition of our research team consisting of Ugandan and non-Ugandan researchers helped to combine outsider with insider perspectives and analysing emerging findings from various angles and perspectives as a team.

Our study followed a strict ethics protocol (Devries et al., 2020). Potential risks were mitigated through regular trainings of researchers, and through a safety and referral plan which included provision of counselling support. Researchers sought participants’ views on preferred times and locations to speak and asked the participants to alert them if they needed to interrupt the discussions, being careful to also listen out for any signs of distress or discomfort. The researchers also took extra care and caution around probing, asking open-ended questions and avoiding direct questions on personal experiences of violence so that the participants were able to maintain control over any personal disclosures.

### 5.1 Qualitative Cohort

In total 17 out of our 36 qualitative core participants indicated that they had experienced kinship care at some point when they were age 0–18 years. We developed and analysed NVivo codes from 6 rounds of fieldwork (2018–2021) and participants biographical narratives from seven rounds of fieldwork (2018–2022). All 17 participants were in informal kinship care when we met them during at least one round of fieldwork. In this process four recurring themes regarding the main characteristics of their informal kinship care arrangements emerged in our data. These are:

• Mitigate poverty and sudden misfortunes
• Ensure continuation of schooling and/or income generation
• Generate and navigate family disputes and ruptures
• Pose and mitigate threats to safety and security

While the biographies of some study participants referred to only one or two of these themes, others encompassed all of them, indicative of the complexity inherent in kinship care arrangements. In Table 1, we provide a very short summary of participants reported kinship care arrangements, as listed above (abbreviated as: *Mitigate Poverty*; *Ensure Schooling/Income*, *Family Disputes/Ruptures; Threat to Safety and Security*). In the ensuing section 6, we discuss and analyse these four recurring themes by paying close attention to young people’s accounts about the main features of the kinship care they are experiencing. Doing so, we sometimes synthesize general findings from multiple participants and sometimes selectively cite respondents. A few participants will be discussed in more detail as short case studies.

**Table 1:** Core Participants who experienced kinship care during ages 0–18 years.

Pseudonym	Gender	Kinship Care Arrangements	Frequency	Main characteristics of kinship care
Ruth	female	Moved between mother’s and grandmother’s house. Father absent	Multiple	Mitigate Poverty Ensure Schooling/Income
Rose	female	Orphan. Lived with: aunt, then two of her sisters, then moved to another uncle and aunt, and now lives with husband	Multiple	Ensure Schooling/Income Family Disputers/Ruptures Threats to Safety and Security
Nakintu	female	Lived with: parents till separation, then grandmother, then father with stepmother, then moved to aunt and uncle, and later to a mother’s friend.	Multiple	Family Disputes/Ruptures Threats to Safety and Security
Aisha	female	Lived with: mother, then aunt and then father	Multiple	Family Disputes/Ruptures Threats to Safety and Security
Tawana	female	Grew up with aunt and uncle, moved in with husband (due to teenage pregnancy), then lived at employer’s home, then moved to cousin’s friend house	Multiple	Family Disputes/Ruptures Threats to Safety and Security
Apio	female	Lived with: Parents (until separation), then moved to father and stepmother, re-located to mother, then grandmother, moved to mother (at her employer’s house), then again grandmother, moved to stepbrother, then to teacher’s home	Multiple	Mitigate Poverty Ensure Schooling/Income Family Disputes/Ruptures
Otim	female	Lived with: mother, then brother, shortly re-located to mother while living with brother (during sickness). Father absent.	Once	Mitigate Poverty Ensure Schooling/Income Family Disputes/Ruptures
Adikini	female	Lived with: Mother, then grandparents. Father died.	Once	Safety and Security Family Disputes/Ruptures
Anna	female	Parents separated. Lived with: mother at grandmothers’ home; then mother and stepfather, after stepfather’s death, lived with grandmother, then relocated to mother’s friend, moved back to mother and new stepfather, later moved to live with paternal aunt, then mother again, then grandmother, then mother, and then moved to maternal aunt, then mother again	Multiple	Mitigate Poverty Ensure Schooling/Income Family Disputes/Ruptures Threats to Safety and Security
Linda	female	Parents separated. Lived with, mother, then grandmother, moved back to father and stepmother, then moved back to mother	Multiple	Family Disputes/Ruptures Threats to Safety and Security
Atala	female	Lived with mother and grandmother. Father absent.	Once	Mitigate Poverty
Musisi	male	Moved between mother, grandparents, aunt and a family friend. Father absent	Multiple	Mitigate Poverty Ensure Schooling/Income Family Disputes/Ruptures Threats to Safety and Security
Paul	male	Lived with father, then grandmother. Mother alive but absent	Once	Family Disputes/Ruptures
Sam	male	Lived with mother (till her death), then father and stepmother, then aunt. His father died while he was living with aunt	Multiple	Family Disputes Threats to Safety and Security
Mugera	male	Father died before his birth. Lived with mother, then grandmother, then aunt and later uncle. Moved btw. these households’ multiple times during childhood.	Multiple	Family Disputes/Ruptures
Kiprotich	male	Parents, grandparents	Once	Family Disputes/Ruptures
Kato	male	Parents divorced. Lived with mother, then aunt, and now father.	Multiple	Family Disputes/Ruptures Threats to Safety and Security

As shown in Table 1 and in our analysis (Section 6), we included study participants under the care of stepparents, when they had either lived with other extended family members before or after they moved into their stepmothers or stepfather’s household. It is also striking that in most cases, young people experienced more than one move and multiple care arrangements, which is not that well reflected or analysed in current literature on kinship care. In the following, we discuss young people’s experiences based on their main characteristics we identified in their informal kinship care arrangements.

## 6 Young people’s informal kinship care experiences

### 6.1 Mitigate poverty and sudden misfortunes

The experiences of several core participants (Ruth, Apio, Otim, Anna, Atala and Musisi) show how kinship care can be strategically used – especially by female caregivers – as an essential means to navigate through multiple socio-economic challenges, sudden misfortunes and poverty. Ruth’s biographical narrative serves as an example. Her parents divorced when her mother was pregnant with her. As the penultimate child, she has five more siblings and to this day she reports that her father hardly provides any support. Her mother, selling food in the streets of Kampala, struggled to take financially care of her six children. Consequently, Ruth was sent by her mother to live with her grandmother for periods of time. This meant that Ruth had to change school multiple times due to her frequent moves between Kampala (where her mother lives) and Luwero (where her grandmother lives). Thanks to the grandmother’s support, she was able (with interruptions), to complete her Senior 4 O-Levels in secondary school. Her final grades were lower than she had hoped for, challenging her aspiration to qualify for a government funded training programme as a nurse. The low socio-economic status of the family required her to work and not study during the holidays (selling roasted maize) to contribute to the family’s income. During the COVID pandemic, her grandmother’s home became an important refuge for Ruth and her siblings. Moving houses meant that the whole family had access to food from the grandmother’s garden during lockdowns. In Ruth’s own words:

*“I came here [grandmother’s home], as life was difficult in town, so my mother decided that we go to the village where we could get free food. Our business was put at a standstill yet we needed to feed every day.”*

Despite constant socio-economic hardships and struggles to make ends meet, Ruth seems to have strong emotional support, stating: “They both [mother and grandmother] loved me so much”. By 2022, Ruth was operating her own business of selling tea, porridge and snacks on a roadside next to her mother’s business, who describes her as hardworking. In the absence of a father and financial support, Ruth’s experience is one of many examples of how female caregivers in economic hardship resort to their family networks to help them raise their children. Similar experiences were shared with us by Otim, Apio or Anna. Although their kinship care differed in frequency or who took care of them, each of them have mothers who would otherwise not have been able to take care of their most basic needs.

The case of Otim is striking, as her situation improved considerably once she moved into the home of her brother, who had accumulated significant wealth through selling charcoal in large bulks. Her parents separated when she was in Primary 5, and her father died soon after. Otim’s mother was left with seven children, which led Otim to miss school temporarily as there was no one to help with school fees. Otim’s circumstances changed promptly when her mother sent her to live with her biological brother, who from then on took care of her. When we asked her shortly after her move to her brother’s house about which home she prefers, she responded:

*“Where I am currently here [brother’s home] works better because it gives me peace.”*

In 2022, Otim was about to complete her A-Levels (Senior 6) in secondary school and had plans and the financial security to enrol at a university studying medicine.

Musisi’s experience, on the other hand, illustrates how extended family networks can also struggle to provide care for all kin children. After his parents separated when he was three years old, he lived at his grandmother’s house together with his four siblings until he reached Primary 4. When his mother re-married, they moved back to live at his stepfather’s house, with only his older brother being raised by his biological father. Musisi’s stepfather took on care of the family but unfortunately died one year into the marriage. His mother, struggling again to financially take care of all children, sent him to live with his aunt. Musisi, however, soon relocated to live at his paternal grandmother’s home, telling us:

*“My aunt asked me to go and start staying at my paternal grandmother’s place (…) I think she was burdened by taking care of us as she had her own children to take care of as well.”*

Eventually, Musisi dropped out of school in Secondary 2 as the extended family could no longer afford to pay for his fees, despite him contributing to costs through small jobs such as digging at the school compound. This was a defining moment for him, as he performed well in class and would have liked to become a doctor. Aged 18, he left his grandmother’s home and started to rent on his own. During our last round of interviews, he worked in construction and was struggling to make ends meet due to a generally difficult economic environment after the pandemic. However, Musisi noted, that he can still rely on his kinship networks as his uncle continues to support him whenever he is in need.

Adikini is an example of how children in kinship care can be disadvantaged within the same extended family networks due to the accumulation of sudden events such as, unemployment, illness and death. It can suddenly change the course of a child’s life, when no extended family support is accessible to help kin children adapt to an extremely difficult situation. At the request of her father, Adikini grew up with her grandfather and step-grandmother, as her mother abandoned her when she was two months. Performing well in primary school, Adikini’s fees and necessities were paid by her grandfather until a series of unfortunate events forced her to drop out of school at Primary 7.:

Joan: *Why did you drop out of school*

Adikini: *There was no more money for school fees, a time reached that my grandfather didn’t have any money. My dad came back but he had also got some problems at his job, when he came back he used all the money he had to build a house and it got finished. We got other problems at home, my uncle died and they spent money during that time. By the time I was supposed to go back to school; my dad had lost his job, my grandfather didn’t have money and he was sick at the time. My grandfather had been bewitched he was almost going to die, he was terribly ill for a long time and all the money that was there was spent.*

Joan: *Did your cousins that you were staying with at your grandfather’s place also drop out of school or did they continue with school?*

Adikini: *They remained in school because we had different fathers. Their father died before mine but their maternal aunt continued paying for them school fees.*

Most of our participants reported that their caregivers struggle in some way to make ends meet and young people’s wellbeing in kinship care appeared to be associated with the wider social support system available to them. Otim fared much better due to her brother’s economic ability and willingness to provide for all her needs, whereas Adikini, Musisi or Ruth’s families faced many economic constraints to provide for their children. Although kinship care helped to mitigate poverty to some extent for Musisi and Ruth in their childhood, they are still trapped in (the same) socio-economic hardships as their caregivers. Both have to cope with and navigate through mounting economic pressures, including coming to terms with compromises they had to make in their educational paths. Inequities faced by their caregivers are relived and reproduced (c.f. Sepúlveda et al., 2022). Besides, our qualitative data shows that involving kin in the care of children due to poverty affects in particular female caregivers with little to no support from their male partners. While we cannot generalize from our small cohort, more research would allow for a more thorough understanding of the gendered dimensions of caregivers’ hardships and the arrangements they have available in their kinship networks to support with childcare. Overall, poverty emerged as a key reason for young people to move into informal kinship care, often repeatedly as the economic strains on mothers can shift. These multiple moves can be planned or sudden in crises (e.g. COVID) and with only very few exceptions (i.e. Otim), most of the young people and their kin continue to experience economic hardships.

### 6.2 Ensure schooling and/or income generation

Linked to the economic reasons, kinship care was also a means of ensuring children’s schooling. Apio’s mother left her with a family member to migrate for labour to be able to provide financial support for her school fees. Ruth’s and Otim’s mothers asked a family member to take them into their homes, knowing that they would also pay for their school fees.

In some cases, not just caregivers but also young people themselves strategically approach extended family members requesting to live with them thereby seeking support for their education. Anna serves as an example. From an early age Anna moved between multiple homes and caregivers due to her parents’ separation and later death of her supportive stepfather. After she had stayed with her grandmother and then a family friend, her mother took her to live with one paternal aunt when Anna was 14 and had just completed Primary 7. Most family members, including her mother, wanted her to join a vocational school after she had completed her primary education. Anna, however, desired to pursue secondary education. After much negotiation, her paternal aunt agreed to pay for her school fees but just one year into secondary school, Anna chose to leave her aunt’s place because she wasn’t getting all the necessary requirements she needed for her education. She approached her grandmother, who eventually agreed to take her in (together with her smaller brother), enrolled her in a school close to her home and started to pay her fees. After a while, her mother, father, uncle and maternal aunt joined her grandmother in financially supporting Anna’s education. Her case is a reminder of how family decisions about kinship care arrangements are often complex, not exclusively made by caregivers and can also be initiated by the child (c.f. Esser et al., 2016). Her story shows how young people can potentially negotiate with supportive family members about their living arrangements and schooling. Even though embedded in a complex web of kinship care arrangements, with the main decision makers not in support of her education, Anna was aware of what her options are, knowing that she had some ability and agency to shape her circumstances (c.f. Van der Kolk, 2014, p. 95).

Putting children through education, or their educational attainment is often seen and used as an important indicator in assessing the wellbeing of children in kinship care (e.g.: Roby et al., 2014, Lachaud et al., 2016, Ariyo et al., 2019). This body of work disregards, however, that children growing up in kinship care can still be well cared for, despite dropping out of school due to a family’s low socio-economic status. Sam’s and Kiprotich’s experiences challenge the common assumption that children in informal kinship care only fare well when they complete their education. Sam grew up with his aunt and uncle. Even though they could not afford to put him through school, they cared for him in a different manner by putting him in charge of small pieces of land or gifting him animals to help him make an income on his own. As with many other participants, his low socio-economic status is not because of kinship care but because of the many economic, social and political conditions affecting the extended family as a whole. Kiprotich is also an example of how young people in kinship care can adapt to dropping out of school – thanks to their extended family support. Growing up with his grandparents he had to leave secondary school as they could no longer afford to pay for his fees, yet he repeatedly told us about his close relationship with his grandparents and fondness of his uncle, also one of his main sources of support. In our final interview, Kiprotich was working in construction, engaging in farming, breeding and selling animals and had already built his first house. During the pandemic he was one of the few participants who did not mention shortages of food or other grievances due to his farming activities and continued extended family support. In both cases, informal kinship care helped them to develop skills for income generating activities.

### 6.3 Generate and navigate family disputes and ruptures

That kinship care is not a static experience, is prone to disputes, and shaped by changing family relationships and power dynamics became evident in several cases. For instance, family disputes led Anna’s mother to disregard extended family member requests to send her to live with her father and instead temporarily moved Anna to a friend’s home. Apio and her sister were moved to a house to be under the guardianship of a housemaid after her father and mother had an argument about who in their kinship networks should look after them. Years later, when Apio and her sister found themselves living with their grandmother during the pandemic, they decided to move out of her home, and rent a place on their own, as she had suddenly too many children to take care of, which made them feel more like a burden than a loved family member.

The case of Mugera is an example of how sudden family ruptures, combined with mistrust and changing relationships can rupture informal kinship care arrangements and young people’s living arrangements later in life. Mugera is the first born and his father died shortly before his birth. When he was still a baby, his mother brought him to live with his paternal grandmother and then soon paternal uncle. He was made to believe by his mother and grandmother that his paternal uncle is his real biological father. It was not until he reached primary education that the family revealed the truth, an experience that disturbed him, as revealed in a conversation with his Ugandan key researcher Brian:

Brian: *How did you feel about suddenly knowing that your biological father had died?*

Mugera: *I felt so sad because they had not told me and they were always lying to me.*

Brian: *How best did you want it to be done?*

Mugera: *They should have told me when I was still young.*

Brian: *Don’t you think it would have created a crisis, as you were too young?*

Mugera: *No because I am the one who even asked them to explain that to me, because people were telling me conflicting information about my father, that he had died. So, I asked them and that is when they told me the truth.*

Brian: *When you got to know the truth, how did you feel about the man they had showed you as your father?*

Mugera: *I did not feel bad because he used to take care of me, and yet the other one had died.*

Brian: *Did you accept him to be your father?*

Mugera: *He no longer cares for me. Right now, when I go home, I am the one who finds money for myself to buy scholastic materials and other school necessities. My grandmother also puts in something for me when she has some cash on her. The other moments when he used to take care of me, he had not married and he had no child. But now he has. Ever since he got a wife, I took on the responsibility for looking for money to support myself. My mother and grandmother just complement when I have failed to find enough.*

In the end Mugera managed to complete his Secondary 4O-Level examinations, thanks to scholarships from non-governmental organizations. Throughout his childhood and later adolescent life, he had an ambiguous relationship with his mother, who was imprisoned shortly after his birth. The reasons for her imprisonment are not known to him. After she was released from prison she gave birth to a daughter and ultimately reached out to him. Mugera describes the relationship as very reserved during that time. He felt loved by his mother but in his words, he was no longer “attached to her”. It was difficult for him to really establish trust and at times he even doubted whether she was his biological mother, in view that she and his grandmother had lied to him about his father. In other interviews, he still notes that he feels very supported by her and cared for. After he had completed his O-Level examinations, and the pandemic started to unfold, Mugera decided to move out of his uncle’s home and stay with his mother. This decision was to the dislike of his uncle, who expected him to help with work. In the end Mugera fell out with his mother as well, moved to his grandmother and then an aunt – having again differences with both. He eventually rented a place with a male friend, but due to repeated disagreements, he had moved back again to his mother’s home when we last spoke to him.

The above affirms Noternan’s (2008) research, showing that kinship care is not void of conflict and competition and in fact part of young people’s daily experience and decision making. Mugera’s case reveals how young people’s experiences of kinship care arrangements is influenced by changing emotions, fluid (not static) relationships, issues of (mis-)trust, specific events, power dynamics, changing household compositions and female caregiver’s life histories (Notermans 2008). During his later adolescence, Mugera moved on to live in a different household whenever a conflict arose. Instead of resolving conflicts with his family members he changed households and living arrangements – with his mother remaining his distant yet constant source of support. We found similar patterns in Apio’s, Anna’s, Paul’s or Rose’ biographical narratives. All told researchers that they chose to leave when they found it difficult to live in their challenging home environment. In short, young people can also experience informal kinship care because of family disputes and ruptures. These informal kinship arrangements might provide needed stability but are not free of conflict, leading in some cases to further moves.

### 6.4 Pose and mitigate threats to safety and security

In some cases, the family disputes were also associated with violence or threats of violence to the young people, and study participants talked of how moves into kinship care resulted from these threats to their safety. It is worth noting, that a recent systematic review of the literature has shown that children in kinship care are not necessarily disadvantaged in terms of safety outcomes when compared to children growing up with their parents or in institutional care (Ariyo, Mortelmans and Wouters, 2019, p. 184). Our qualiative cohort pointed to both, how kinship care can expose some of our participants to violence, but also how kinship networks intervene to mitigate threats to saftey and security for kin children. In most cases, our participants have been either removed from violent kinship cargivers by other extended family members, or decided themselves to leave.

Nakinto, for example, lived with both parents until she was in Primary 1 when they separated. Together with her siblings, she was first taken to live with her grandmother until her father remarried. She moved back into her father’s new home with her stepmother, who severely mistreated her, involving physical violence in the form of beating. When a neighbour cautioned Nakintu’s stepmother to stop beating her step-children, she resorted to other measures of abuse, such as overloading her with work in the mornings so that she would reach school late on a daily basis. Nakintu further told us:

Nakintu: *She sometimes used to blackmail me to my father and this could hurt me, when she is jazzing with her friends all they talk about is me and my mother which used to annoy me very much. I always to her to insult me as much as she wished but she shouldn’t involve my mother at any time.*

Rehema: *What was she talking about your mother that used to annoy you so much?*

Nakintu: *She calls my mother an adulterous woman who even abandoned her children and went on. Not knowing that there must be a reason for her going, I believe no one wishes to leave her children behind.*

The stepmother then told Nakintu’s grandmother that she would poison Nakintu if she was not moved to another household. Her aunt consequently made her stay with another uncle, where she lived until Secondary 3. Nakintu enjoyed living at her uncle’s place for a couple of years until she experienced emotional and physical violence at his home as well. She was blamed for dating her cousin’s boyfriend (her uncle’s biological daughter) and carrying out abortions which she claims are false accusations. Eventually, Nakintu moved back to her father’s home. She seems to have coped with and adjusted well to this sudden change. According to her key researcher, she comes across as a confident and cheerful young woman. When we spoke to her during our final round of interviews, Nakintu was in a boarding school for a vocational training programme in tailoring, following her dream of becoming a fashion designer.

Also Rose, an orphan, independently decided to move out of her older sister’s home due to conflict and abuse. In her words:

*“(…) time came when I couldn’t stay with my sister anymore because she used to insult me about all the things she provides for me publicly. I made up my mind to go back to the village and I had stayed there for just a short while then my other sister came and picked me. So I am currently living with her.”*

For Rose, her decision to change her kinship care arrangement came at a cost she was aware of and willing to undertake. She knowingly compromised her education, paid by her oldest sister, for a safe and loving home environment. She was aware that, escaping the abuse by her older sister meant dropping out of secondary school in year 2; requiring her to help her other sister with childcare, cooking and household chores.

Adikini is an example of how multiple traumas and experiences of violence can also coincide during and with kinship care. Adikini’s mother left when she was two months old. They stay in touch but in Adikini’s words have a “reserved” relationship. She mainly grew up with her grandfather, who she is fond of, and her step-grandmother, with whom she had some conflict in the past but the relationship improved later on. Her father died when she was fifteen years old. At the age of nine Adikini was raped and badly injured by a stranger who had just moved into the community. She dropped out of school at Primary 7 (see section 6.2 for details), and started to work as a housmaid in Kampala where she faced again violent treatment by her female and sexual advances by her male employer. Having been raped as a child, her father’s murder, and the abuse she suffered as a housemaid were both deeply traumatic. When we had first met her, Adikini was seventeen years old, lived with her step-grandmother and grandfather, and had already a two-year-old daughter. The father of the child, for whom she had only good words for, provided some support but was away to take care of his sick mother when we first met her. Adikini shared with us how and in what ways her step-grandmother ignored all her traumatic childhood experiences and still mistreated her, by trying to beat her, denying Adikini and her daughter food, or insulting her in front of strangers. It reached a point in which Adikini attempted suicide:

*“I was tired of the situation so I went to Dekabusa trading center and bought poison. I was going to take the poison and die because I thought dying was easier than living through such life. I was about to take it and my cousin found me, he removed it from me and poured it and told me not to do it but I was going to do it and die and leave this situation*

After her cousin had saved her life, her grandfather, together with her uncle and aunt had a conversation with her step-grandmother. The relationship has improved since then. When we met Adikini two years later, she was in good terms with her step-grandmother. However, in the end Adikini did become estranged from her extended family, including her beloved grandfather. After she had separated from her daughter’s father, she moved to another village with a new boyfriend and gave birth to a baby boy. She almost entirely cut off contact with her grandparents and extended family and only occasionally gets in touch with one of her brothers. Her new partner appears to be very protective and controlling of her social ties and relationships, but she still puts a lot of hope into the relationship.

The above cases show that young people’s experience in kinship care strongly depends on the extended kinship structures as sources of support available to them. Nakintu’s and Adikini’s extended family members were eventually able to protect them from further abuse. For some young people, these experiences of abuse and conflict in kinship care led them to cut off family ties. For others, like Nakintu, they maintained close ties with their kinship carers, and with their mothers, recognising the difficulties they had experienced when raising children. Even Adikini, who initially had a lot of resentment towards her biological mother, got closer to her again during her late adolescence. It was her mother who helped taking care of Adikini’s daughter during the pandemic, and later nursed her back to health after the birth of her son. This opens up many unexplored questions on the emotional and psychological dimensions of informal kinship care and what kind of support is and should be available to children and young people in these settings.

## 7 Concluding discussion

As the role and depiction of a ‘family’ including ‘mothers’ and ‘fathers’ are constantly debated and renegotiated due to changing political, economic and social conditions, norms and context, so is the nature and function of informal kinship care. The experiences of our qualitative cohort have shown that informal kinship care networks are essential for children, especially for those from a low socio-economic background and with caregivers struggling to make ends meet. As a cultural institution and practice, informal kinship care changed significantly since colonialism, from a traditional family model aiming at strengthening wide social and political ties, to a socio-economic necessity to cope with increasing poverty and sudden misfortunes. Extended families have become an important and indispensable safety net to help Ugandan caregivers navigate through the many economic and political hardships they have to deal with on a daily basis. Neoliberal interventionism, capitalism and globalisation all played a part in this development (Tamale, 2020). The present political and economic conditions have far-reaching social implications and consequences for caregiving arrangements in Uganda, requiring attention and more targeted support from governmental and non-governmental actors, aid- and development agencies. Our analysis brought to light the many kinds of pressures (financial, emotional, or health related), families have to come to terms with and how these are manifested in informal kinship care arrangements.

***The mitigation of poverty and sudden misfortunes***, emerged as a key reason for young people to move into informal kinship care, often repeatedly as the economic strains on mothers can shift. These multiple moves can be planned or sudden in crises (e.g. COVID) and with only very few exceptions, most of the young people and their kin continue to experience economic hardships. Our analysis further shows how informal kinship care is a strategic tool, mostly by single or divorced mothers, to ensure their children are well looked after; stay enrolled in school, and to overcome multiple economic and social pressures. More research and support are needed in this regard to provide a nuanced understanding of the gendered dimension of kinship care and social injustices. In short, what does informal kinship care tell us about the adaptive capacity of mothers and fathers in situations of economic hardship, sudden misfortunes and a general difficult economic and political environment? There is still little knowledge about the different pressures faced by female and male caregivers to send their children away including the type and frequency of support they provide to their children when not in their care. Most importantly, strategies are needed to support female caregivers who involuntarily have to send their children to live with extended family to make ends meet.

How kinship care helped to ***ensure schooling and/or income generation*** confirms existing research concluding that, how children fare in informal kinship care, depends on the wealth available to them in their extended family networks (Roby et al., 2014, Ariyo et al., 2019). However, we caution against the general trend to use ‘education’ as one of the main indicators for children’s wellbeing in informal kinship care, as our cohort has shown that kin children can still fare well and receive emotional and other support, such as help with income generating activities, despite dropping out of school.

Our third finding, that kinship care could ***generate and navigate family disputes and ruptures*** brought to light, that in contrast to children raised by their biological parent(s), kin children have to adapt from an early age to social variability, mobility, the changing nature of relationships and how to cope and deal with sudden misfortunes or disappointments. While children in informal kinship care tend to be approached and portrayed in the literature as having a rather passive role in where and how they live or deal with their situation, the accounts of some of our core participants suggest the opposite. More attention should be given in research and practice to the agency and leeway children have themselves in kinship care (c.f. Esser et al., 2016). This further extends to their coping strategies and how they navigate through disappointments and disputes. Our cohort has shown that this can range from moving between family members to seeking and receiving support from relatives to engage in income generating activities. More knowledge is needed to better understand how adaptive strategies differ among boys and girls in this context.

Our fourth finding was that kinship care could both ***pose and moderate threats to safety and security of children***. We found that young people in our cohort who lack extended family support (financial as well as emotional), faced more challenges when it comes to completing their education or coping with abuse they have been exposed to. While Nakintu had the much-needed support to escape her stepmother’s abuse, it took much longer for Adikini’s family to finally intervene. Future research could help us to deepen our understanding of how neglect in informal kinship care influences young people’s relationships and social behaviour during later stages in their lives – also from a gendered lens. More generally, our study gives rise to questions on how children who are subject to mistreatment in informal kinship care could be better supported and what kind of mechanisms should be in place to protect them from further abuse? If anything, their cases bring to light that adequate support systems to assess caregivers and children’s needs in kinship care are currently not in place. Overall, it is important to acknowledge that informal kinship care networks can expose children to abuse but also play an essential role in mitigating threats to children’s safety and security.

Considering the high prevalence of informal kinship care, more recognition and support is needed from various actors whose responsibility it is to ensure adequate childcare arrangements and to protect children’s rights. This requires moving away from standardised and institutionalized care to individualised, culturally-sensitive and context-specific support. In practice, this could for instance translate into supporting a more flexible mode of schooling for children in informal kinship care, considering that kin children change schools more frequently and scholarships are often tied to one specific institution. Every time a child moves, they can potentially fall through school- or any other protection systems. More generally, the role of schools but also local community networks and groups could be further explored to help provide financial or emotional support to informal kinship caregivers and their kin children. This could include the establishment of support groups within communities, or specific networks where children and caregivers can receive counselling services (if needed), or simply connect with others facing similar challenges. While targeted financial support to caregivers may seem plausible to help them cover costs associated with caring for an additional child or children; such initiatives can pose several challenges in practice. They would require informing communities about the programme, helping them access financial support, avoiding misuse of funds and ensuring that financial support is used for the benefit of the child.

To conclude, we have shown how informal kinship care occurs within the legacies of colonialism, shaped and strained by specific social, political and economic contexts. As a persistent practice of childcare, informal kinship care continues to challenge western notions of family units and caregiving which prevail in global health programmes. More detailed, context-specific and meaningful understanding of children’s care and living arrangements is needed in programming and policy. This further extends to paying more attention to the role children play in navigating family relationships, in some cases, across multiple homes and villages as they grow up. All of this has serious implications for health, social protection, education programming and policy for children.

## 8 Funder

Medical Research Council.

Grant number: MR/R002827/1.

See: https://gtr.ukri.org/projects?ref=MR%2FR002827%2F1.

## Author Statement

Simone Datzberger conceived the kinship care study, performed the main analysis, reviewed the literature, and drafted the manuscript. Jenny Parkes and Amiya Bhatia participated in the study design, provided critical comments, and helped revise the manuscript. Rehema Nagawa, Joan Ritar Kasidi, Brian Junior Musenze and Karen Devries commented critically on the manuscript and provided input into the analysis. Karen Devries and Jenny Parkes are co-PIs on the CoVAC study. All authors have read and approved the final version of the manuscript and agree with the order of presentation of the authors.

## Declaration of competing interest

The authors declare that they have no known competing financial interests or personal relationships that could have appeared to influence the work reported in this paper.

## Data Availability

The data that has been used is confidential.

## Footnotes

1 The Baganda are the largest ethnic group in Uganda, belonging to the Kingdom of Buganda the largest of the country’s traditional kingdoms.

2 Cited from: https://www.worldbank.org/en/country/uganda/overview accessed 9.2.2023.

3 Ibid.

4 See: https://data.unwomen.org/country/uganda, accessed 9.2.2023.
