# Supplementary material for: Young people’s experiences of informal kinship care in Luwero, Uganda
Source: Child Youth Serv Rev. 2024 Apr;159:107527. doi: 10.1016/j.childyouth.2024.107527 (PMC11750426; doi:10.1016/j.childyouth.2024.107527)
Supplement: Supplementary data 1 [file mmc1.pdf]

Annex 1

Annex 1: Overview of CoVAC qualitative data collection 2018-2022	
**Fieldwork 1**<br><br>October – December 2018	○ Face to Face interviews with 36 core participants (2-3 hours)<br>○ Community walks with 36 core participants (1-2 hours, followed by 1-2 hour interviews)<br>○ 33 caregiver interviews (1-2 hours)<br>○ 6 FGDs (1-3 hours)<br>○ All data translated, transcribed and coded in Nvivo<br>○ Biographical summaries and fieldnotes were created
**Informal stay in touch calls**<br><br>May – June 2019	○ Short phone conversations (10-20 minutes)<br>○ Not transcribed or coded but information from call was added to biographical summary
**Fieldwork 2**<br><br>October – December 2019	○ Face to face interviews with 35 out of 36 core participants (2-3 hours)<br>○ 9 teacher interviews (1-2 hours)<br>○ 8 stakeholder interviews (1-2 hours)<br>○ 4 FGDs (1-3 hours)<br>○ All data translated, transcribed and coded in Nvivo<br>○ Biographical summaries and fieldnotes were updated
**Formal stay in touch calls**<br><br>May – June 2020	○ Longer phone conversations (30-45 minutes) with 34 out of 36 participants<br>○ All data translated, transcribed and coded in Nvivo<br>○ Biographical summaries and fieldnotes were updated
**Fieldwork 3**<br><br>October – December 2020	○ Face to face or phone interviews with 35 core participants (1-2 hours)<br>○ 9 peer interviews (1-2 hours)<br>○ 10 teacher interviews (1-2 hours)<br>○ All data translated, transcribed and coded in Nvivo<br>○ Biographical summaries and fieldnotes were updated
**Formal stay in touch calls**<br><br>May – August 2021	○ Longer phone conversations with 35 out of 36 participants (30-45 minutes)

	○ All data translated, transcribed and coded in Nvivo<br>○ Biographical summaries and fieldnotes were updated
**Fieldwork 4**<br>October – December 2022	○ Face to face interviews with 36 core participants (2-3 hours)<br>○ Face to face interviews with 34 caregivers (1-2 hours)<br>○ 7 peer interviews (1-2 hours)<br>○ 7 teacher interviews (1-2 hours)<br>○ All data translated, transcribed and coded in Nvivo<br>○ Biographical summaries and fieldnotes were updated
